# Management of Central Nervous System Cavernomas: An Experience of the Department of Neurosurgery at the Ibn Tofail Hospital, Mohammed VI University Hospital

**DOI:** 10.7759/cureus.33028

**Published:** 2022-12-27

**Authors:** Yassine El Allouchi, Hajar Hamadi, Lamia Benantar, Khalid Aniba

**Affiliations:** 1 Neurological Surgery, Ibn Tofail Hospital, Mohammed VI University Hospital, Marrakech, MAR

**Keywords:** cavernous malformations, microsurgery, radiosurgery, epilepsy, cavernomas, central nervous system

## Abstract

Introduction: Central nervous system cavernomas are congenital vascular anomalies posing a challenge not only in their diagnosis but also in their therapeutic management. The latter depends largely on their natural evolution and specifically their hemorrhagic potential.

Material and methods: This paper reports the experience of the Department of Neurosurgery at the Ibn Tofail Hospital, Mohammed VI University Hospital at Marrakech in the management of central nervous system cavernomas from January 2011 to December 2018. We collected and analyzed epidemiological, clinical, radiological, therapeutic, and evolution data from 16 cases of central nervous cavernomas using a pre-established sheet.

Results: Sixteen cases of cavernomas were treated in a period of eight years; 14 patients had cerebral cavernomas and two had brainstem cavernomas. The sex ratio was 1.66 with a male predominance, and the mean age of our patients was 42 years. The clinical presentation was dominated by epileptic seizures. Cerebral MRI was performed on all our patients. In all 16 cases, the cavernomas were solitary with the majority being supra-tentorial (13 cases) and bleeding was apparent on imaging in one case. Microsurgery was performed in 13 cases, while three patients benefited from stereotactic radiosurgery. Complete resection was obtained in all patients and pathology examination showed a radio-histological correlation in 87.5% of cases. The overall evolution in operated patients was favorably marked by neurological improvement in 87.5% of cases, deterioration in 6.2% of cases, and no clinical improvement in 6.2% of the cases.

Conclusion: Early diagnosis coupled with macroscopically complete resection and long-term follow-up with MRI are all crucial steps to ensure the proper management of central nervous cavernomas, especially considering their risk of recurrence.

## Introduction

Cavernomas, also known as cavernous hemangiomas, are congenital vascular anomalies described for the first time by Virchow in 1863. They represent 5% to 10% of cerebral vascular malformations and are usually diagnosed post-mortem during autopsy or as an incidental finding on imaging in 0.4% to 0.8% of cases [1,2]. They can be localized anywhere in the central nervous system. They are characterized by their potential for growth with the risk of hemorrhagic rupture, usually accompanied by moderate but recurrent bleeding.

This pathological entity, angiographically occult, benefited greatly from the development of medical imaging techniques specifically MRIs, as well as microsurgery and stereotactic radiosurgery. The positive diagnosis of this pathology has been made considerably easier, which explains the increase in its frequency. However, the true incidence and natural history of cavernomas are still not fully understood, posing a challenge and controversy regarding their management and therapeutic indications.

We present a retrospective study of 16 cases of cavernomas of the central nervous system; we highlight our experience in the surgical management of this pathology using either microsurgery or stereotactic radiosurgery.

## Materials and methods

We performed a retrospective study on a series of 16 cases of cavernomas of the central nervous system who were hospitalized at the Department of Neurosurgery, Ibn Tofail Hospital - Centre Hospitalier Universitaire (CHU) Mohamed VI University Hospital at Marrakech for a period of eight years, spanning from January 2011 to December 2018. The different parameters and data pertaining to these cases were obtained from the medical records of the patients at the Neurosurgery and Anatomic Pathology Departments of the CHU Mohamed VI University Hospital, Marrakech. The local ethics committee did not request an institutional review board (IRB) since this is a retrospective study without identifying information. 

A pre-established operating sheet was used (after patient and hospital consent was obtained) to gather epidemiological, clinical, paraclinical, therapeutic, and evolution information. These parameters were collected and analyzed using a simple computation of the statistical data.

## Results

Amongst our 16 patients, we counted 10 males and six females with a sex ratio of 1.66, and a mean age of 42 years. Fourteen of our patients had cerebral cavernomas (including the cerebellum and brainstem localizations) while the remaining two had intramedullary spinal cord cavernomas (Table 1).

**Table 1:** Epidemiological data

Localisation		Brain	Spinal cord	total
Mean age (years)		36	48	42
Sexe (number of cases)	Male	08	02	10
	Female	06	00	06

The delay between onset and admission ranged from 15 days to three months in 54.5% of cases. Epilepsy was the most common clinical sign leading to diagnosis followed by neurological deficit and headaches (Table 2).

**Table 2:** Revealing clinical signs

Revealing clinical signs	Number of cases	%
Headache	01	6,2
Epilepsy	10	62,5
Neurological deficit	05	31,2

**Figure 1:**
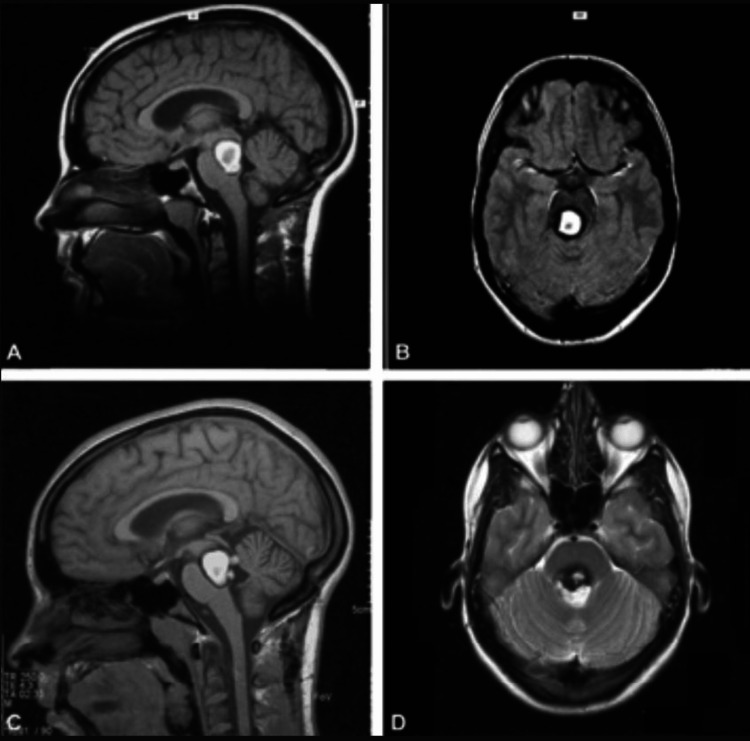
Cerebral MRI showing a brainstem cavernoma A,C: Brain MRI in sagittal planes showing a posterior mesencephalic lesion in hypersignal flair consistent with a brainstem cavernoma; B,D: Brain MRI in axial planes showing a brainstem cavernoma in hypersignal flair.

The image of a cavernoma was found either on a CT scan in apparent cases, or on a hemosiderin MRI sequence. Of the 16 cases of solitary cavernomas, hemorrhage was present in three patients. In the other 13 cases, no bleeding was visible on imaging (Figure 1).

The mean size of the cavernomas was globally 22,3 mm (21.9 mm in cerebral cavernomas and 16.6 in brainstem cavernomas).

Of the 14 cases of solitary cerebral cavernomas, 13 were supratentorial with a frontal localization in five cases and parietal in three cases. Only one patient had a infra-tentorial cavernoma (6,2%) and two patients had spinal cord cavernomas (Table 3).

**Table 3:** Localization of the cavernomas

Localization	Number of cases	Percentage %
Supratentorial	13	81,5
Infra-tentorial	01	6
Spinal cord	02	12,5

In our series, 13 patients underwent microsurgical treatment while three were treated using stereotactic radiosurgery. The therapeutic indication was based on the prevention of the hemorrhagic risk, particularly at the brainstem and spinal cord levels, and for the treatment of a plausible epilepsy in minimally hemorrhagic or non-hemorrhagic forms.

**Figure 2:**
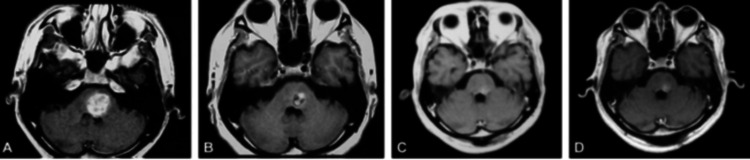
Evolution of a brainstem cavernoma treated with stereotactic radiosurgery after 36 months of follow-up. A: MRI imaging of the brain in axial planes in T1 sequence showing a brainstem cavernoma after stereotactic radiosurgery; B: Follow-up MRI imaging in T1 axial planes showing the brainstem cavernoma at 12 months post stereotactic radiosurgery; C: Follow-up MRI imaging in T1 axial planes at 24 months post stereotactic radiosurgery showing a significant volume reduction; D: Follow-up MRI imaging in T1 axial planes 36 months post stereotactic radiosurgery showing a stable lesion with no volume increase.

Post-operative follow-up was marked by an improvement of the neurological state in 14 of our patients (87.5%); neurological deterioration in one patient was treated with a standard surgical approach (6.2%), and there was no clinical improvement in one patient treated with stereotactic radiosurgery (6.2%) (Figure 2).

Only one patient presented with a reccurence of the cavernoma (6.2%) which was diagnosed on a follow-up cerebral MRI performed one year post-operation.

## Discussion

The exact prevalence of cavernomas in the general population is still uncertain. But due to diagnostic advances and the widespread use of MRI in clinical practice; their incidence increased with a prevalence of 0.4% to 0.9% [1-2].

Cavernous angiomas can be found at any age but the majority of cases are diagnosed before the age of 60 with an age range of 30 to 50 years of age, and both sexes are equally affected (sex ratio=1) [1,3]. In our series, the mean age was 42, with a male predominance (62.5%).

Due to their variability in size, location, and hemorrhagic risk, they can cause a great spectrum of clinical symptoms. The most commonly encountered clinical symptoms are epilepsy followed by neurological deficit and headaches [1,3-5,6] which concords with our findings.

MRI, though not the gold standard for diagnosis, is currently the most performing imaging tool, not only to pose a diagnosis but also for post-operative follow-up; MRI was performed on all our patients.

The characteristic aspect of MRI is that of a heterogenous lesion in T1 and T2 hypersignal, surrounded by a hyposignal zone on T1 and T2 sequences, with no mass effect or perilesional edema. This typical image makes the positive diagnosis of cavernomas straightforward. Anatomopathological analysis, mandatory after surgery, confirmed the diagnosis in all cases treated surgically. The supratentorial localization is more common than the sub tentorial (80% vs 20% of all cases). Our results conform with this anatomical distribution. The annual rate of bleeding varies between 0.2% and 3.1% per patient per year, with a maximum rate of 5.7% in sub-tentorial localizations [1,6-9].

The location of the malformation is a predictive factor of hemorrhagic accidents. The bleeding risk is higher with subtentorial and deeply located cavernomas [7,10]. This risk is further increased after an initial episode [10]. Prospective hemorrhage rates have been reported between 0.7% and 6.5% per lesion-year with an annual rate of rebleed of 4.5% at a mean interval of 12 months between the initial bleeding and the second episode [10].

Our series includes three cases of cavernomas with bleeding, two of which were solitary cerebral cavernomas and one medullary. Thus, the calculated annual risk of bleeding (0.01% per patient per year) seems extremely low, probably due to the underevaluation of hemorrhagic accidents [8,11-13].

The surgical decision has to be thoroughly discussed. It requires vast experience and perfect knowledge of surgical anatomy, and more precisely, the knowledge of surgical approaches with minimal risk. The strategy of the surgical approach is guided by the findings of MRI [3]. A conservative management is preferred in asymptomatic or mildly symptomatic patients, while complete resection is the only healing option for surgically accessible symptomatic or hemorrhagic lesions. The results obtained in our series concerning the treatment of epilepsy in operated patients concord with the results of the literature [3,13-15]. A total resection of the cavernoma was performed in all operated patients.

The indication of stereotactic radiosurgery in the treatment of angious cavernoma is the object of controversy. Analysis of its risk/benefit ratio shows that radiosurgery should not be regarded as an alternative to surgery. furthermore, its value with regard to decreasing the hemorrhagic risk has not been proven. In the radiosurgical literature, the annual rebleeding rate after radiosurgery is 8%-10% for the first two years and 0.8%-4.5% thereafter. These rates correspond to the bleeding incidence in studies on the natural evolution of cavernomas [15]. Nevertheless, in cases of partial resection with a persistent risk of hemorrhage and in patients who are poor surgical candidates, it can be a useful complement [13-15]. 

It is argued that an established diagnosis of epilepsy resistant to pharmacotherapy justifies the indications of surgical treatment. Nevertheless, the favorable evolution of seizures (a decrease in frequency or disappearance of seizures) in a great number of patients on long-term pharmacotherapy urges caution in the indication of surgical treatment. The latter should be preferably reserved for hemorrhagic forms [8,11,13,14].

Physical rehabilitation is a key aspect of the management of neurological deficits secondary to cavernomas. It improves surgical results and increases the chances of full neurological recovery [1].

The morbidity and mortality of this pathology are highly dependent on the neurological state prior to surgery [1,5-7,10]. The evaluation of permanent sequelae is fixed at three years postoperatively. And it is generally admitted that the long-term neurological state is closely similar to the postoperative findings [13].

Follow-up using MRI is performed at a frequency that depends largely on the complete or incomplete character of the surgical resection.

## Conclusions

Cerebral cavernomas represent 5% to 13% of central nervous system vascular malformations affecting all age groups with a net surge from the second to fourth decades of life. Their etiopathogeny has recently been linked to genetic mutations. Although CT scan has been a great diagnostic tool, MRI remains far more efficient in allowing for an early diagnosis. The choice of treatment options, be it conservative or surgical, depends on the clinical presentation of the lesions and their hemorrhagic risk. The best treatment option remains resection for surgically accessible lesions and hemorrhagic lesions with radiosurgery being reserved for deeply located cavernomas. Therefore, an early diagnosis and adequate management are key for a favorable surgical outcome.
